# Development of a Mobile Application of Internet-Based Support Program on Parenting Outcomes for Primiparous Women

**DOI:** 10.3390/ijerph18147354

**Published:** 2021-07-09

**Authors:** Xilin Li, Yao Zhang, Ziwen Ye, Lingling Huang, Xujuan Zheng

**Affiliations:** Health Science Centre, Shenzhen University, Shenzhen 518060, China; xy_l1991@163.com (X.L.); zhangyao@szu.edu.cn (Y.Z.); y673817550@126.com (Z.Y.); huanglingling@szu.edu.cn (L.H.)

**Keywords:** M-health, mobile phone, application, primiparous women, maternal self-efficacy, social support, postpartum depression

## Abstract

Primiparous women usually experience various parenting problems after childbirth that have negative effects on the well-being of mothers and infants. Although e-Support technology could provide an innovative and easily accessible intervention approach, mobile-phone interventions remain limited for Chinese primiparous women. Therefore, a new mobile application (APP) called the “Internet-based Support Program” (“ISP”) was designed, incorporating the self-efficacy theory and the social-exchange theory for Chinese first-time mothers to improve their levels of maternal self-efficacy (MSE), social support, and satisfaction, as well as to reduce their postpartum depression symptoms. The research was conducted to develop and optimize the “ISP” APP for new mothers via a theory-, evidence-, and person-based approach. Five modules of “learning forum”, “communication forum”, “ask-the-expert forum”, “baby home forum”, and “reminder forum” were included in the APP to meet various parenting needs of first-time mothers; and its contents and functions were validated by the experts and primiparous women. The majority of participants gave positive feedback on the APP’s perceived ease of use and usefulness. The “ISP” APP was the first designed for Chinese primiparous women, and a multicenter randomized controlled trial (RCT) will be conducted to measure its effectiveness on parenting outcomes.

## 1. Introduction

Having a baby is indeed an event that irrevocably alters a woman’s life [1]. Maternal-role transition will bring tremendous challenges for first-time mothers, who need to learn various parenting knowledge and skills and adjust to the new household relationship [2]. Owing to the little parenting experience, primiparous women after childbirth usually suffer from various parenting problems, which have negative effects on the physical and mental well-being of mothers and infants [3,4,5]. Maternal self-efficacy (MSE), as a significant indicator of parenting outcomes, is the belief women hold about their capability to organize and perform the different parenting tasks [6]. Women with a high level of MSE are identified to have positive parenting outcomes [7]. However, research found that Chinese primiparous women had a moderate level of MSE and were frequently confronted with many parenting problems, i.e., unsuccessful parenting tasks and negative mother–child attachment [8].

Evidence supports that many factors affect MSE and the main factors influencing MSE are postpartum depression (PPD) and social support [5]. Studies demonstrated that, compared with women in Western countries, Chinese primiparous women were more likely to encounter PPD, because of the overwhelming maternal-role expectations in Chinese culture and the nerve-racking and vulnerable relationship with their mother-in-law [8,9]. Research found that women with PPD were prone to have worse physical and mental status which negatively impact on their MSE levels [5,10,11]. By contrast, studies undertaken in different countries consistently found that social support positively affected MSE and maternal mental status [12,13,14]. Chinese first-time mothers were reported to acquire insufficient support after delivery and extremely lack the informational and appraisal supports from the health professionals on various parenting tasks [8].

In order to improve the parenting outcomes for new mothers, some traditional face-to-face interventions were undertaken and proved to have an effect [9,15]. For example, the effects of educational program oriented interpersonal psychotherapy were assessed via a RCT study in China. Women in the experience group received two face-to-face education trainings of a total of three hours, as well as a telephone follow-up in two weeks postpartum. Compared with women in control group, new mothers in study group had significant higher scores of MSE and social support, and a lower score of PPD at three months postpartum [9,15]. However, the promotion of these traditional face-to-face intervention approaches was challenged by the huge number of Chinese new mothers and the insufficient Chinese health professionals [16]. Therefore, the alternative intervention methods with easy access, innovation, and effectiveness need to be designed. Evidence found in comparison with the traditional face-to-face intervention and internet interventions can transmit more tailor information through multimedia, reach larger target research population, and assure more anonymity [17].

Nowadays, the fast-speed broad-band Internet offers new interactive multi-media experiences, which are currently used in different resources to increase maternal confidence focusing on parenting knowledge, skills, or behaviors [18]. Several researchers have suggested that the internet has great potential for delivering parenting interventions in an accessible way [18,19,20,21]. For instance, blogging was found to improve new mothers’ well-being, as they feel more connected to the world outside their home through the Internet [22]. Research have reported internet-based parenting programs had some significant positive effect on parenting skills, mental well-being [18,19,22], and children outcomes [23], but the evidence for this was limited due to the lack of experimental designs [19]. The narrative review found that the benefits of using online technologies were increased self-esteem, perceived social support, and increased opportunity for self-disclosure; however, the harmful effects were also reported as increased exposure to harm, social isolation, depression, and cyber-bullying [24].

In 2020, approximately two-thirds of Chinese population were reported to use the internet [25], and 99% of them accessed the internet by mobile devices [26]. Thus, mobile application (APP) could provide a promising platform to conduct intervention for Chinese primiparous women. To the best of our knowledge, no RCTs by APP intervention have been conducted in China on parenting outcomes for primiparous women. Therefore, a mobile application of “Internet-based Support Program” (“ISP”) on primiparous women’s parenting outcomes was developed in the research. It is the first designed with documented evidence base and medical professional involvement for Chinese primiparous women. The research was conducted to develop and optimize the “ISP”APP for new mothers to improve their parenting outcomes, social support, and satisfaction, as well as to reduce their PPD symptoms

## 2. Materials and Methods

A new mobile application, called “ISP” APP was developed in China to promote mothers’ capability to complete various parenting tasks and improve mother-infant attachment by enhancement of MSE and social support, thus improving their psychological well-being and satisfaction. The theoretical framework of “ISP” APP incorporated the Bandura’s self-efficacy theory [27] and the social-exchange theory [28]. According to the self-efficacy theory, MSE could be affected by four major elements, including previous parenting experience, vicarious parenting experience, verbal persuasion from others, and maternal physical and mental status. In terms of social support, it could be conceptualized by structural and functional components. The structural social support was regarded as an informal social network from family members and friends, and formal social network from health professionals. The functional social support has included informational, instrumental, emotional, and appraisal support. Based on the social-exchange theory, the structural social networks of women could be built, and the various kinds of functional supports could be provided form the health professionals and others through the “ISP” APP. The research likewise has approved that the core components of parenting interventions should assure the appropriate MSE and social-support levels [11].

A theory-, evidence-, and person-based approach was used to develop and optimize the “ISP” APP. Firstly, a mixed method study was conducted to explore main parenting needs and to identify barriers and facilitators to use of digital technology in primiparous women via the semi-structured interview (*n* = 9) and the questionnaire survey (*n* = 344). In the interview, some questions were asked, such as “Please share your story about being a new mom”; “Do you have any parenting problems? What your needs or expectations in terms of parenting?” and “What do you think about the online parenting support?”

**Figure 1:**
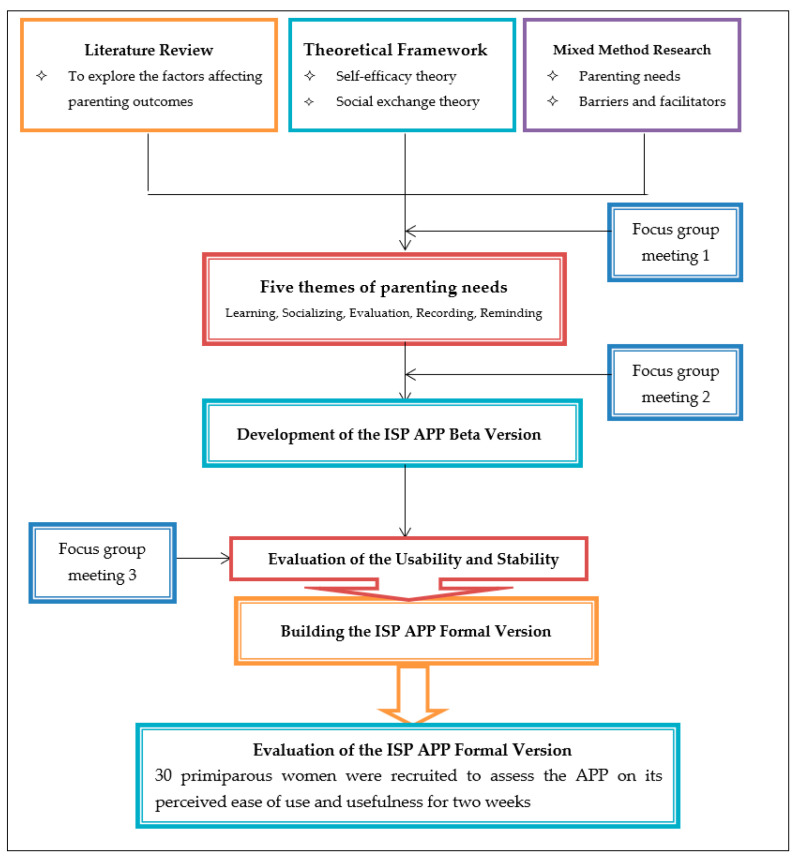
Stages in the process of developing the “ISP” APP.

Five themes of maternal parenting needs were identified, namely learning requirements, socializing requirements, evaluation requirements, recording requirements, and reminder requirements. Furthermore, two focus-group meetings were attended by seven parenting experts and five technical experts to discuss the significant components of APP and its detailed contents to fulfill the above parenting needs and functions. Based on the literature review, theoretical framework, mix research results, and expert consulting, the five core components of “ISP” APP were formed, namely “learning forum”, “communication forum”, “ask-the-expert forum”, “baby home forum”, and “reminder forum”. After the software development was completed, the other five experts were invited to conduct functional tests on the APP beta version and give feedback on the usability and security evaluation, so as to improve the usability and stability of the APP. Stages in the process of developing the “ISP” APP lasted about one year, from January to December 2020, and they are summarized in Figure 1.

## 3. Results

### 3.1. The Development Environment and Interface Design of the APP

Based on the established mobile health online intervention, the technical personnel and researchers worked together to develop the “ISP” APP system. An internet company in the People’s Republic of China conducted the technical development and the technical maintenance of this APP. The “ISP” APP program is supported on both android mobile phones and iPhones. The Android version uses the Eclipse integrated development environment and Android SDK; while the iPhone version uses the Xcode integrated development environment and iOS SDK. The website background management system was written by PHP/JavaScript language with Eclipse integrated development environment.

**Figure 2:**
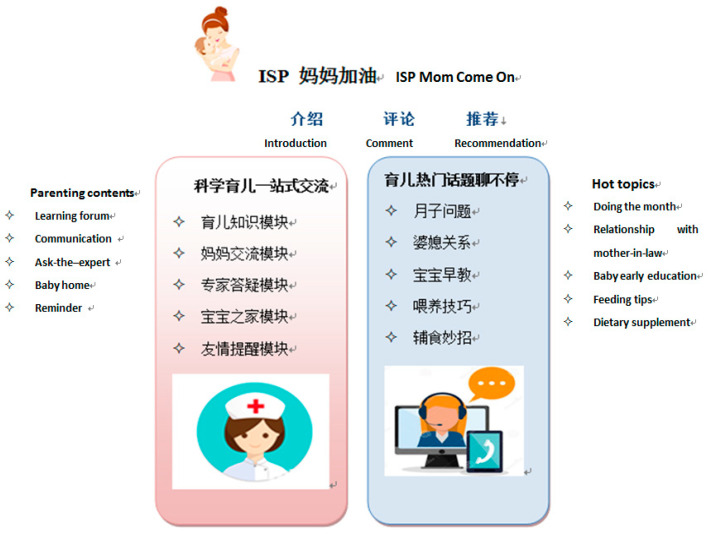
The interface design of “ISP” APP.

Considering the perceived ease use of APP, the interface design is simple and intuitive, and the language is easy to understand, as there is a greater use of pictures and videos (see Figure 2). Considering the perceived usefulness of the APP, the contents of parenting were rich to meet various needs of first-time mothers. After installing the APP, women need to submit their application of ISP. Once their applications were approved, the researchers would coach them on how to log in and use each component of APP with the original username and password, which could be changed by women later. The adherence of women was assessed by their recorded frequency and duration of the logins. Technical assistance can help women via telephone or email on the weekdays.

### 3.2. Core Components of the “ISP” APP

**Figure 3:**
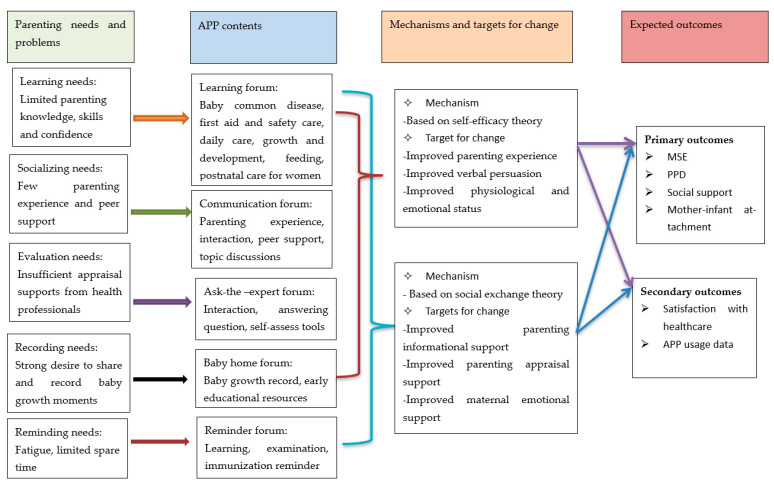
Logic model for “ISP”APP to support new mothers with parenting and mental well-being.

The five core components of “ISP” APP were identified to meet primiparous women’s various parenting needs. Logic model for “ISP” APP to support new mothers with parenting and mental well-being was shown in Figure 3. The “learning forum” offers parenting knowledge and skills to first-time mothers via multimedia resources to fulfill women’s educational needs. The contents of this forum comprise six parts: (a) intervention and nursing of common baby health problems, including pathological jaundice, neonatal umbilicus, eczema, colic, thrush, constipation, diarrhea, fever, cough, asthma, urticaria, hand-foot-mouth disease, infantile pneumonia, infant’s exanthema subitum, herpes pharyngitis, otitis media, urinary tract infection, pillow bald, and mosquito bites; (b) first aid and safety care, such as head injury, burn and scald, falling injury, cardiopulmonary resuscitation for baby, airway foreign body obstruction trachea, electrically damaging, and drowning; (c) daily care of infants, i.e., indoor environment preparation for infants, sleeping, dressing, changing diapers, bathing care, urinary and fecal problems, crying care, umbilical cord care, skincare, trimming nail, nasal care, and private care; (d) baby growth and development, such as the curves of baby height and weight, baby head circumference and shape, bone and tooth development, sensory and perceptual development, major motor development (lifting head, rolling over, sitting, crawling, standing, walking, etc.), fine motor development, language development, social behavior development, baby touch, and parent–child interaction and attachment; (e) breastfeeding and bottle feeding, i.e., breastfeed principles, feeding posture, frequency and duration of feeding, burping, spitting up, and other common feeding problems; and (f) postnatal care for women, such as maternal role transition, postnatal physical and psychological changes, postnatal exercise, and do’s and do not’s of “doing the month”.

The “communication forum” supplies the platform to meet women’s social needs where new mothers can share their parenting tips, exchange parenting feelings and experiences, seek peer resonance, belonging and support. In this forum, some hot discussion themes are offered for first-time mothers, such as maternal-role transition, new family relationship, “doing the month”, asking for supports, dealing with the relationship with mother-in-law, and how to continue breastfeeding after the maternity leave.

In the “ask-the-expert forum”, there are effective interactions between parenting experts and new mothers. Health professionals can give prompt feedback and evaluation of supports to women about their asked common parenting questions and posted parenting problems within one day. Common tools of self-assess for babies, such as BSID (the Bayley Scales of Infant and Toddler Development) [29] and Chinese baby’s normal growth curve program [30], are also supplied in the component.

The “baby home forum” meets women’s recording needs, which provides women with the opportunity to make baby growth and development record and share their feelings and comments on maternal role. Various multimedia resources of Early Child Development (ECD) are also included in this APP component. The “reminder forum” sets up realistic task setting and reminds users to facilitate fulfilling parenting activities, such as the APP learning reminder, the postnatal physical and mental examination reminder, the questionnaire filling reminder, and baby immunization reminder.

### 3.3. Evaluation of the “ISP”APP

All eligible women in maternity ward in Shenzhen City were invited by researchers to participate if they met the inclusion criteria: (1) being postnatal women aged 18 years or over, (2) with a healthy infant, and (3) having the ability to respond. Exclusion criteria were (1) women whose baby was seriously ill or died and (2) women with a severe physical or mental disease. The researcher contacted and approached eligible postnatal women as early as possible after childbirth, gave them an information sheet, and answered their questions about the research. Maximum variation sampling was used to recruit participants that would best represent the diversity of the women.

In total, 30 primiparous women were recruited to use and assess the APP in two weeks. The sociodemographic and clinical data of these participants are summarized in Table 1.

These respondents were varied in their age when they giving birth, educational level, occupation, family income, mode of delivery, baby gender, and the version of the mobile-phone system. Maximum variation sampling was beneficial to gain greater insights into a phenomenon by looking at it from all angles and helped the researcher to identify the usability and feasibility of the APP.

The APP evaluation data of perceived ease of use and perceived usefulness among 30 new mothers are shown in Table 2. The total APP usage time of 30 participants was approximately 600 h in two weeks. Higher than 90% of women thought the APP could download easily; and about 90% of them evaluated the layout of APP was reasonably designed. All of participants thought that the parenting contents of the APP were useful to the user and were easy to understood. The majority of women (≥90%) gave the positive feedback of the APP operation. No significantly statistical difference on the evaluation and usage time of the APP was found among women’s subgroups comparisons of different sociodemographic and clinical levels

### 3.4. Optimization of the “ISP” APP

According to the users’ feedback, the guideline was added in the “reminder forum” to help women download, install and navigate the various sites within the “ISP” program. Based on the women’s concerns and questions in the “ask-the-expert forum” and “communication forum”, more parenting knowledge and information was added in the “learning forum”, such as how to make positive, affirming, loving comments to the infant; display a range of facial expressions; provide safe environment; play with infant appropriately; and recognize signs of health problems, describe infant symptoms clearly, and seek appropriate healthcare. The participation of these users helps to optimize the content and design of the APP, making it much more useful, attractive and easy to use for first-time mothers, thereby promoting their understanding of the APP.

## 4. Discussion

The research was conducted to develop and evaluate the “ISP”APP for new mothers to improve their parenting outcomes, social support, and satisfaction, as well as to reduce their PPD symptoms. This study indicated the “ISP” APP had a significantly high level of usability and acceptability, and could help primiparous women to address gaps in various parenting information and supports after childbirth.

According to the Technology Acceptance Model (TAM), one of the most influential models of technology acceptance, there are two primary factors affecting an individual’s intention to use new technology: perceived ease of use and perceived usefulness [31]. Perceived ease of use refers to “the degree, to which the user expects the target system to be free of effort”; and perceived usefulness means the individual’s “subjective probability, that using a specific application system, will increase his or her performance within an organization context” [31]. The participants in this research were generally comfortable with use of the technology and thought the “ISP” APP could use easily or feel easy to learn, which was consistent with the previous study of developing the “Brain-Fit” APP [32]. However, some studies had reported that their participants lacked familiarity with digital technology which regarded as a common barrier to use [33,34,35]. This could be due to the relatively young age of primiparous women in comparison to elder persons in previous studies. Moreover, in order to improve the APP’s perceived ease use in the current research, the interface design is simple and intuitive; and the language is easy to understand, as there is a greater use of pictures and videos to make it more interesting and brief.

In the present study, all participants indicated that the “ISP” APP was useful to the user, thus positively influencing women to use the new technology [31]. Some factors attributed to the users’ positive feedback on the “ISP” APP. Firstly, the person-based approach used [32,36,37] allowed for the integration of current evidence and the perspectives of end users with the APP in a systematic and iterative manner to assure fully understand new mothers’ various parenting needs. Secondly, extensive qualitative and quantitative evidence provided by a mixed-method approach could explore and map the potential barriers and facilitators of new mothers to the use of parenting APP. The effective user engagement could improve women’s evaluation of the APP’s perceived usefulness [32]. The key strength of the research was that we conducted the structured iterative development and optimization process of the APP to make ensure its positive usability and acceptability. However, the potential limitation of the study is that a majority of participants were younger women. It could affect the user’s responses on acceptability of digital technology and may cause over estimation of the familiarity of the target population with APP. Furthermore, there are only two focus-group meetings on the APP development, owing to the time and financial limitation, and were thereby less than is typically viewed as optimum [38]. Moreover, even though no significantly statistical difference on the APP usability evaluation was found among the subgroup comparisons of different educational levels, it could be caused by the small sample size in the study. Therefore, women with less education should be better represented in the further research sample that evaluated the APP. Additionally, about one-third of Chinese population in 2020 cannot access the internet [25]. Especially for those mothers who were in more remote rural areas or less familiar with the online technology, their problem of APP application could not be simply resolved through the concise APP guideline, without available face-to-face support and follow-up. Therefore, the digital literacy and access to the internet of those women were strongly recommended to be provided by Chinese government for the widespread use of APP.

## 5. Conclusions

The aim of the “ISP” APP used is to promote mothers’ capability to complete various parenting tasks and improve mother–infant attachment by enhancement of MSE and social support, thus improving their psychological well-being and satisfaction. Based on the literature review, theoretical framework, mixed-research results, and expert consulting, the five core components of “ISP” APP were formed, namely “learning forum”, “communication forum”, “ask-the-expert forum”, “baby home forum”, and “reminder forum”, to meet the various parenting needs of first-time mothers. The majority of participants gave positive feedback on the APP’s perceived ease of use and usefulness. The “ISP”APP was the first designed for Chinese primiparous women, and a multicenter RCT will be conducted to measure its effectiveness on parenting outcomes.

## Figures and Tables

**Table 1:** Socio-demographic and clinical data of participants (*n* = 30).

Variables	Frequency	Percentage (%)
**Age When Giving Birth**		
20–25	10	33.3
26–30	12	40.0
31–40	8	26.7
**Education**		
Middle school or lower	6	20.0
High school	9	30.0
University/college or higher	1	50.0
**Occupation**		
Professional	6	20.0
Skilled	9	30.0
Unskilled	7	23.3
Unemployed	8	26.7
**Family Income (RMB Per Month, Per Person)**		
<4000 yuan	9	30.0
4001–6000 yuan	11	36.7
>6000 yuan	10	33.3
**Mode of Birth**		
Normal vaginal birth	15	50.0
Assisted birth	5	16.7
Caesarean section	10	33.3
**Infant Gender**		
Girl	14	46.7
Boy	16	53.3
**Mobile-Phone System**		
Android	17	56.7
Apple (iOS)	13	43.3

**Table 2:** The APP evaluation data of perceived ease of use and usefulness of participants (*n* = 30).

Headings	Items	Evaluation/Usability Data Percentage (Frequency)		
		Good	General	Bad
Download	1. The APP could be properly installed on the device	97% (29)	-	3% (1)
	2. The APP could be installed easily	93% (28)	7% (2)	-
	3. The APP icons could be on the phone after installation	100% (30)	-	-
	4. The researchers could give your useful instructions	93% (28)	7% (2)	-
Layout design	1. The launch page could be reasonable	90% (27)	10% (3)	
	2. The launch page could be helpful to the user	97% (29)	3% (1)	-
	3. The navigation could be accurate and intuitive	100% (30)	-	-
	4. The registration and login page layout could be reasonable	87% (26)	13% (4)	-
	5. The menu bar could be reasonably designed	100% (30)	-	-
	6. The category page could be reasonable	90% (27)	10% (3)	-
Contents	1. The parenting contents could be useful to the user	100% (30)	-	-
	2. The contents could be met your various parenting needs	90% (27)	10% (3)	-
	3. The combination of the pictures and texts could be acceptable and clearly	90% (27)	10% (3)	-
	4. The size of pictures and words could be appropriate	97% (27)	3% (3)	-
	5. The contents could be easily understood	100% (30)	-	-
	6. The experts feedback could be promptly	90% (27)	7% (2)	3% (1)
	7. The communication subjects could be attractively	87% (26)	13% (4)	-
	8. The “reminder forum: could be useful to the user	83% (25)	17% (5)	-
Operation	1. The APP running could be smoothly	93% (28)	7% (2)	-
	2. Videos could be playing normally	100% (30)	-	-
	3. Switching speed could be acceptable between different forum interfaces	90% (27)	10% (3)	-
	4. Repeated switch between background and foreground could be normal	93% (28)	7% (2)	-
Duration		2 weeks		
Frequency		1.43 h per day per person		
Total usage time		600 h		

## Data Availability

The data presented in this study are available on request from the corresponding author. The data are not publicly available due to privacy restrictions.
